# Managing Students’ Creativity in Music Education – The Mediating Role of Frustration Tolerance and Moderating Role of Emotion Regulation

**DOI:** 10.3389/fpsyg.2022.843531

**Published:** 2022-04-13

**Authors:** Lei Wang, Na Jiang

**Affiliations:** ^1^School of Music, Liaoning Normal University, Dalian, China; ^2^Changchun Humanities and Sciences College, Changchun, China

**Keywords:** creativity, emotion regulation, negative emotion, frustration tolerance, artificial intelligence

## Abstract

Artificial intelligence (AI) era challenges the use and functions of emotion in college students and the students’ college life is often experienced as an emotional rollercoaster, negative and positive emotion can affect the emotional outcomes, but we know very little about how students can ride it most effectively to increase their creativity. We introduce frustration tolerance as a mediator and emotion regulation as a moderator to investigate the mechanism of creativity improvement under negative emotion. Drawing on a sample of 283 students from professional music colleges or music major in normal universities, we find that negative emotion are generally associated with a lower creativity, while frustration tolerance can mediate the relationship between negative emotion and creativity, but these effects depend on the emotion regulation. Cognitive reappraisal exerts a negative effect on the relationship between negative emotion and creativity, while expressive suppression has the opposite effect. Our study contributes to the literatures on student’s emotions and creativity in music education and to the emotion regulation literature.

## Introduction

In the new era of artificial intelligence (AI), big data and cloud computing, more and more attention has been deployed to emotion, because emotion is fundamental to human experiences influencing our daily activities including cognition, communication, learning, and decision making and it has been a very crucial factors influencing students’ creativity in college to facilitate the multiple creation (Brodin, 2018), for example, music improvisation. In reality, college music education called for the training of students’ creativity, so did the society and parents, because music needs more creativity in creation. When students have more creativity, they can create wonderful work, which can bring more for music students for their development and growth. With the reform of the education, the students’ creativity has not been effectively improved. Faced with the pressure in life and study, there are a lot of negative emotions from student, which will inhibit students’ creativity (Chen et al., 2019) in music education. As we all know, for centuries, psychologists have tried to understand and define emotions because of its importance in affecting creativity. The most widely recognized basic emotions proposed by Ekman were happiness, sadness, fear, anger, surprise, and disgust. Humans express and recognize emotions in multiple ways in which the expression of emotions is one of the heavily studied areas. So it is necessary to study this topic in the view of emotion and find ways to regulate the influence on students’ creativity in college life.

However, the current literatures elaborated the influence of students’ creativity in the four aspects: *first*, the relationship between student and their parents can bring negative mood for their behavior, such as family background, economic situation, which have an awful influence on student’s mood (Rossetto and Chapple, 2019; Cheong et al., 2021; Colson et al., 2021). *Second*, the relationship between student and teachers exerts special influence on the creativity in the view of imbalance, for example, their conflict can affect the mood and behaviors (Chang et al., 2016; Soh, 2017). *Third*, the internal control and coordination can influence student’s creativity in learning process. *Fourth*, the boundary conditions need more explanation among the negative emotion and creativity and the current moderators focus on internal and external conditions, such as emotion experience, emotion labor and mental elasticity (Stephenson et al., 2018). *Fifth*, the black box between negative emotion and creativity is not open because the extant studies paid more attention to the negative effect of negative emotion on creativity, but seldom focus on how to change negative emotion into creativity and the path received little attention.

In order to answer the above concerns, we introduce frustration tolerance as an essential path between emotion and creativity. But the effects of frustration tolerance on students’ creativity and the moderating role of emotion regulation have received little attention (Runco et al., 2017; Gál et al., 2021). Frustration tolerance is an individual’s ability to tolerate adversity and unmet needs in order to maintain normal lives. In college, the level of individual frustration tolerance is affected by many subjective and objective factors, and is also related to the individual’s past experience, especially in music education (Wu et al., 2014). If the degree of frustration exceeds the range of self-tolerance, it is bound to destroy the normal performance of self-function, and then hinder their life adaptation. Therefore, it is indeed necessary to study the relationship between frustration tolerance and creativity.

Based on cognitive appraisal theory and using data of 283 students from professional music colleges or music major in normal universities, we contribute to emotion research in creativity literature by showing the important impact of negative emotion on student’s creativity. Our study provides insights into how students’ emotion regulation enables themselves to bounce back from bad mood in music education. Students who tend to suppress their emotions will be more unable to improve creativity. In contrast, students who tend to reappraise situations may be able to perceive negative emotions accurately, thereby activating problem-focused coping mechanisms and make creative behaviors. Knowing students can use frustration tolerance to bounce back from negative emotion is not only vital for student’s creativity, but also for future development of students by using emotion regulation.

## Literature and Hypotheses

### Taking an Emotive View on Creativity

There is a mount of research focusing on the determinant factors influencing students’ creativity (Glover, 1980; Ashby et al., 1999). The creativity literature highlights that factors contributing to the improvement of students’ creativity remain poorly understood, especially under the circumstances of negative emotion, and that there is still a significant gap in our understanding of how to improve students’ creativity (Furnham et al., 2011; Zhao et al., 2019). We do not focus on traditional views which suggest that students’ creativity is brought about by external factors, such as college’s rules, student-teacher relationship or even social atmosphere that students have little or no control, or that creativity depends mainly on student’s ability to create something innovatively. Instead, we draw on the recently developed emotive approach to student’s creativity, which suggests that the student’s creativity is not simply a matter of internal abilities (Kolyvas and Nikiforos, 2021), but that more subjective and psychological reasons contribute to creativity (Oliveira et al., 2021). By focusing on psychological attributes, the emotive view provides a powerful perspective on student’s creativity, as it explains why some students with negative emotion have high creativity, while others with positive have low one (Isen et al., 1987). Therefore, we introduce cognitive appraisal theory, which believes that emotion is a reaction process in which individuals perceive environmental events as beneficial or harmful and students can react to the events to regulate their emotion and find psychological ways to deal with these matters.

### Creativity

Creativity is the ability to combine or connect elements to form a new relationship (Rahimi and Shute, 2021). Individuals with creativity have psychological traits such as curiosity, adventure, challenge and imagination in their emotions. Creativity explains the psychological process of transcending existing experiences, breaking through habitual limitations and forming new ideas in new situations (Bereczki and Kárpáti, 2018). It is not restricted by conventional rules and can flexibly apply experience to solve problems. Students’ creativity is a kind of ability with which the individual can function flexible, unique, progressive characteristics in the supportive environment through the process of thinking to produce divergent views, endow things unique, novel meaning, and gain the results not only make themselves, but also make others satisfied. For students majoring in music, their creativity is of great value to their own creation, especially improvisation (Falavarjani and Yeh, 2018). And individual creativity is diverse and variable, and should not be limited to the innovation idea itself, but should also include the generation of innovative ideas, content, implementation, promotion and development to ensure that innovative ideas can be implemented effectively in the music creation (Torrance and Myers, 1970; Zhou and George, 2001; Rossetto and Chapple, 2019). So we conceptualize the creativity as the ability of individuals to produce innovative and feasible ideas for solving problems. Torrance and Myers (1970) divided creativity into four dimension, and they are originality, which means the degree of innovation in a method of solving a problem, constructive diversity, which means the number and diversity of methods proposed to solve problems, analytical power, which means the degree of details of the method of solving problem and feasibility, which refers to the degree to which the method proposed to solve the problem can be applied in real life.

### Frustration Tolerance

In Zhang’s Dictionary of Psychology, frustration tolerance is defined as an adaptive ability of individuals to avoid abnormal behavior and continue efforts when confronted with blows and setbacks, which causes individuals to withstand difficulties, setbacks and failures when encountering setbacks. It is an individual’s ability to adapt to frustration and setbacks. Frustration tolerance reflects an individual’s ability to endure possible failures and continue to participate in challenging work, which can affect an individual’s willingness to take risks and his response to failure (Clifford, 1991). Frustration tolerance ensures that students are able to cope effectively with stress, adapt to daily challenges, recover from disappointment, adversity and trauma, and develop a sense of purpose. This plays an important role in solving problems and getting along with others for college students (Posner et al., 2005). Clifford (1984) proposed *the Theory of Constructive Failure*, which clarifies and reevaluates the functions of failures and setbacks. If the importance of success is blindly emphasized, it will deprive individuals of the opportunity to feel failure and frustration, which individuals cannot learn from Roth et al. (2021). Frustration tolerance in this study is defined as an individual’s ability to endure failure and frustration in learning, and to persevere in the face of difficulties.

### Negative Emotion

Emotional theory divided emotion into emotional tones, such as happiness, anger, sadness and emotional dynamics, such as emotional intensity, duration, and recovery time (Russell, 1980). Early studies focused on the tone of emotions and paid more attention to the nature of emotions (Chen et al., 2019), but recent studies tend to focus on the kinetic energy of emotions (Liu, 2022), such as the stimulation of emotions and the process of emotional calm. Yela et al. (2019) emphasized the importance of individuals’ active construction and evaluation of situations. He believed that individuals undergo a cognitive evaluation process between the occurrence of emotional stimulation and emotional response, which is why the same event can produce different emotional responses for different individuals. Watson and Tellegen (1985) took the degree of individual subjective feeling of pleasure as the benchmark for differentiation and divided emotions into positive and negative ones. If the feeling is favorable to oneself, it will produce positive emotions, if the feeling is unfavorable to oneself, it will produce negative emotions. Negative emotion is a kind of external behavior of escape, resistance and other physiological unpleasant.

### Emotion Regulation

Emotion regulation is defined as a kind of process that influences the emotional intensity over time and influences whether, when, and how we experience and express positive or negative emotions (Fredrickson, 1998; Gross, 1998, 2013; Gross and Thompson, 2007). Gross (1998) proposed a process model of emotion regulation, which has five sequential strategies, and they are situation selection, situation modification, attentional deployment, cognitive change (cognitive reappraisal) and response modulation (expressive suppression). Individuals may regulate their emotions by using different strategies (Gross and John, 2003). In our study, we follow Gross and John (2003) in defining, analyzing, and measuring emotion regulation as the tendency to habitually use a specific emotion regulation strategy.

We choose to focus on cognitive reappraisal and expressive suppression, two of the most-established and important emotion regulation strategies (Gross and John, 2003; Webb et al., 2012; Gross, 2013). Gross and John (2003) proposed the two emotion regulation strategies, and studied their different influences on emotion, cognition and social behavior. Lazarus and Alfert (1964) pointed out that cognitive reappraisal is a type of cognitive change, which involves explaining a potential emotional withdrawal situation in a non-emotional way, thus changing the impact of the current emotion. On the contrary, Gross (2013) pointed out that expressive suppression is a type of response modulation, and takes place later in the process to inhibit ongoing emotional expressions, which requires not only cognitive strategies, but also long-term use of these strategies to monitor and inhibit the expression of emotions.

Focusing on these two types allows us to contrast between an antecedent- and a response-focused emotion regulation strategy. We believe that cognitive reappraisal and expressive suppression are two crucial emotion regulation strategies that are particularly relevant in student’s college learning (Roth et al., 2021) as they, respectively, relate to emotional experience and expression and can affect student’s behaviors (Sun et al., 2022). We expect that how they deal with the student’s negative emotions will be different to some extent.

### Hypotheses Development

It is obvious that individual students with positive emotions can improve their positive effects in emotion, cognition and action (Watson, 2018). However, negative emotions restrict student’s ability to think and act, and even show a state of self-protection, and inhibit the expression of creativity. Negative emotions such as depression and fear can lead students to have no confidence, disappointment, self-doubt, and make individual lose the courage to try, or even hinder creative ideas, which may inhibit creativity (Yeh et al., 2019). The higher the degree of positive emotion, the better individual creativity. Conversely, the higher the degree of negative emotion, the worse individual creativity. Huang (2019) discussed creativity from the perspective of neurophysiological responses and argued that negative emotions would reduce the stimulation of knowledge and interfere with information integration. Negative emotions tend to be conservative in the way individuals deal with problems and reduce creativity (Averill, 2000). Negative emotion can limit individual’s thinking and reduce the ability of association and analysis, as well as lack of motivation, which makes it more difficult for individuals to exert their creativity when they have negative emotion. All of these can stop music students to create new productions. Watson and Hubbard (1996) pointed out that when individuals are in negative emotions, they would try to isolate themselves from stressors, and even deny themselves. Based on this, the following hypotheses are proposed in this study:

Hypothesis 1. There is a negative relationship between the negative emotion and creativity.

The theory of constructive failure affirms the positive influence of setbacks and frustration. Tolerance is produced by the combination of an individual’s attitude toward failure, external goals and intrinsic motivation (Pi et al., 2019; Shirish et al., 2021). Frustration tolerance is acquired through learning and cultivation, and it is a sign of good adaptation and mental health. In addition, Oliveira et al. (2021) also believed that there are obvious individual differences in frustration tolerance, even if the same person, when facing different frustration events, their physical and mental reaction and frustration tolerance are not the same.

When individuals face the impact of external events or its internal needs are not satisfied, they can tolerate and adjust the negative emotions brought by setbacks, solve the dilemma with a positive attitude and promote the improvement of creativity (Liu et al., 2020). Researchers believe that frustration tolerance should be a necessary ability for individuals, and it can be more perfect after acquired training and learning. In the face of negative emotions, frustration tolerance can help individuals to identify the problem, seek the root of the problem, promote individuals to be positive, and then enhance their creativity (Pi et al., 2019). In college education, it is normal for student to encounter setbacks and difficulties, and their negative emotions often exist. Only students with strong tolerance of setbacks and frustration will not be discouraged when they occasionally meet adversity (Shi et al., 2021). They can improve their own emotions and mentality by facing up to setbacks and enhance their creativity. Therefore, we proposed the following hypotheses:

Hypothesis 2. There is a positive relationship between frustration tolerance and creativity.
Hypothesis 3. Frustration tolerance mediate the relationship between negative emotion and creativity.

As shown above, cognitive appraisal theory believes that emotion is a reaction process in which individuals perceive environmental events as beneficial or harmful and this emotion can facilitate individuals to cope with the events by taking emotive measures (Lazarus, 1991). Emotion regulation is the ability to detect their own and other people’s emotions, and then appropriately deal with emotions and adjust their own thinking and action (Snyder et al., 2019). Cognitive reappraisal is “a form of cognitive change that involves construing a potentially emotion-eliciting situation in a way that changes its emotional impact” (Gross and John, 2003: 349). As an antecedent-focused type of emotion regulation, it can help individual to deal with upcoming emotional events. Previous research shows that cognitive reappraisal is of help to student and assist them to reframe setbacks or obstacles as opportunities, because cognitive reappraisal is associated with better psychological health, optimistic views, and wellbeing (John and Gross, 2004; Gross, 2013), all of which may prevent students from suffering negative emotions and encourage them to recognize themselves and discover the source of negative emotions (Morina et al., 2018). As we all know, negative emotions can weaken the ability of cognitive improvement, thus receding the creativity.

In other words, individuals with high cognitive reappraisal can properly express their own emotional responses, and adopt flexible strategies to adjust their own emotions and deal with the emotional responses of others. Individuals can properly use the influence of emotions to strengthen their tolerance for setbacks. It’s good for creative thinking, motivation and even distraction. Catanzaro (1990) pointed out that when individual is in an emotional state generated by stimulus, he can self-perceive the emotion, and try to adjust the emotion by behavioral and cognitive changes to adapt to the environment or events. Gross (2013) believed that individuals who often use cognitive reappraisal strategy can improve their emotional and interpersonal functions and enhance their creativity.

Expressive suppression is “a form of response modulation that involves inhibiting ongoing emotion-expressive behavior” (Gross and John, 2003: 349). It is a response-focused strategy, meaning that this strategy can change the public display but not the internal experience of the emotion. According to cognitive appraisal theory, Individuals using suppression frequently leads to high emotional exhaustion, which in turn strengthens the bad effect of negative emotions. Furthermore, Schroder (2021) found that expressive suppression decreases student’s satisfaction on daily work, which in turn strengthens intentions to give up the current work, thus reducing the deeper thinking in production and students’ giving up halfway (D’Mello and Duckworth, 2019; Pujol, 2019). In addition, students who suppress their emotions are also seen as desolate and lack of confidence for some matters, which can attenuate the creativity as a result of negative emotion (Shi et al., 2021). Although the individuals who often used “expressive suppression” strategy could effectively adjust their emotions, they could not reduce the influence of negative emotions, especially on music creativity.

“Emotional Alchemy” proposed by Cooper and Sawaf (1997) believes that individuals with high cognitive reappraisal can quickly deal with their negative emotions and focus on solving problems. Therefore, when individuals encounter negative emotions, they can adjust their emotions and transform the power originally used to fight against negative emotions into energy to stimulate creativity. In challenging situations, the individual expressive suppression would affect their curiosity and problem-solving ability, thus affecting creativity. This results in the following hypotheses:

Hypothesis 4. Cognitive reappraisal negatively moderates the relationship between negative emotion and creativity.
Hypothesis 5. Expressive suppression positively moderates the relationship between negative emotion and creativity.

**FIGURE 1:**
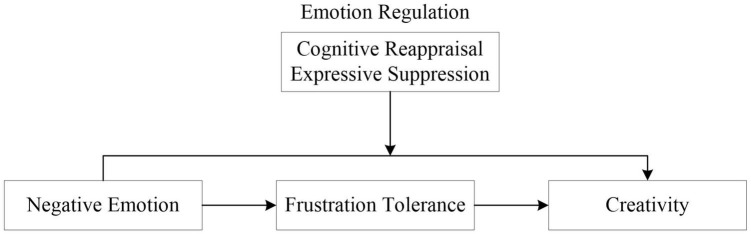
The conceptual model.

Figure 1 depicts the conceptual model of the research.

## Materials and Methods

### Sample and Data Collection

We used a sample of students from professional music colleges or music major in normal universities in Northeast of China. We contacted the 13 teachers in 7 colleges or universities and asked them to help distribute the survey questionnaire, through which we collected data on emotion regulation, negative emotions, frustration tolerance, and creativity and student demographics. Although emotion regulation, creativity and frustration tolerance may vary across an individual’s university grade – differences are found in freshman, sophomore, junior and senior stages (Gross, 2013). But we believed it remain stable over this survey period.

In total, we contacted 1,360 students who agreed to participate in this survey. We delivered 1,360 questionnaires and collected 513 samples. After deleting the questionnaires with missing key items we obtained 283 complete, usable questionnaires, giving us a response rate of 20.8%. We evaluated non-response bias (283 vs. 230) by testing for differences in means for each variable (*t* > 0.05) and compared the former and latter questionnaires (177 vs. 106) and found that non-response bias (*t* > 0.05) doesn’t exists.

### Variable Measurement

#### Creativity

Recently, divergent thinking has become an important index to measure music student’s creativity. TTCT (Torrance Tests of Creative Thinking) proposed by Torrance and Myers (1970) is widely used to measure creativity. TTCT divides creativity into language and graphics, and these division can give special explanation for music improvisation. In music education, language and graphics can teach students special knowledge and give them extra hints in their created work (Watson, 2018; Colson et al., 2021), so TTCT (Torrance Tests of Creative Thinking) is often used in testing students’ creativity in education, especially in music, arts, and other humanities majors. Therefore, TTCT is very suitable and right for measure creativity for music education. TTCT uses four characteristics to measure creativity (see Supplementary Appendix Table 1). They are (1) originality (the degree of innovation in a method of solving a problem), (2) constructive diversity (the number and diversity of methods proposed to solve problems), (3) analytical power (the degree of details of the method of solving problem), and (4) feasibility (the degree to which the method proposed to solve the problem can be applied in real life). The Cronbach’s alpha for this measure was 0.84.

#### Frustration Tolerance

We borrowed and revised 18 measurement items from School Failure Tolerance Scale (SFT) developed by Clifford (1984) to measure frustration tolerance. After factor analysis, we deleted three items with low factor loading (see Supplementary Appendix Table 2). The Cronbach’s alpha for this measure was 0.91.

#### Negative Emotion

We measured negative emotion using Fisher’s (1997) eight-item scale (see Supplementary Appendix Table 3). The Cronbach’s alpha for this measure was 0.86.

#### Emotion Regulation

We measured cognitive reappraisal using Gross and John’s (2003) six-item scale (see Supplementary Appendix Table 4). The Cronbach’s alpha for this measure was 0.89. Expressive suppression was measured using Gross and John’s (2003) four-item scale. The Cronbach’s alpha was 0.80.

#### Control Variables

We chose student’s age, gender as control variables because they are regarded as influencing factors for creativity (Lai et al., 2019; Kolyvas and Nikiforos, 2021) and emotion regulation (Liu, 2021). We also controlled for the Big Five factors (i.e., neuroticism, agreeableness, extraversion, conscientiousness, and openness to experience) and measured these using John et al.’s (1991) Big Five Inventory (BFI) (see Supplementary Appendix Table 5). The Cronbach reliability values were 0.92 for neuroticism, 0.94 for agreeableness, 0.93 for extraversion, 0.90 for conscientiousness, and 0.92 for openness to experience and the total Cronbach reliability values of Big Five is 0.96 after deleting the low factor loading items. All variables are valued by Likert-5 level.

#### Common Method Bias

Common method bias may appear when both independent and dependent variables are derived from the same source at the same time (Podsakoff et al., 2003). This is the case in this study. Nevertheless, we tested for this in two ways. First, we used Harmon one-factor analysis without rotation and found that the first factor can only explain 37.71% of variance and there was no single factor, but five ones. Second, we used a latent method variable in our model to examine what influence common method variance might have on our results. The loadings of this latent construct on the variables in our model were relatively low (between 0.07 and 0.39), and the differences in estimates for our main variables between the models with and without the latent variable were small (average deviation = 0.03, maximum deviation = 0.11), and the correlation coefficients with other variables (see Table 1) range from −0.04 to 0.13, suggesting that common method bias was not a big concern.

**TABLE 1:** Correlation matrix.

	1	2	3	4	5	6	7	8	9	10	11	12	13
1. age	1												
2. gender	0.00	1											
3. neuro	−0.09	0.05	1										
4. agree	0.11	0.12*	0.23**	1									
5. extra	−0.03	−0.03	0.25**	0.32**	1								
6. consc	0.09	0.12*	0.34**	0.19**	0.33**	1							
7. open	0.11	0.11	0.24**	0.29**	0.40**	0.28**	1						
8. creat	0.02	0.04	0.15**	0.21**	0.14*	0.23**	0.22**	1					
9. ft	0.01	0.11	0.29**	0.38**	0.37**	34**	0.68**	0.22**	1				
10. ne	−0.09	0.05	0.23*	0.21**	−0.31**	−0.23**	−0.25**	−0.14*	0.28**	1			
11. cr	0.06	−0.01	0.12*	0.20**	0.27**	0.20**	0.22**	−0.16**	0.21**	0.25**	1		
12. es	0.08	−0.02	0.25**	−0.26**	0.23**	0.28**	0.28**	−0.20**	−0.33**	0.20**	−0.19**	1	
13. grade	0.02	0.03	−0.03	0.00	0.03	−0.01	0.01	−0.01	−0.02	0.04	−0.018	0.05	1
14. mv	−0.07	−0.03	0.06	0.07	0.09	0.04	0.17*	0.11	0.24*	0.05	0.10	0.06	0.09
Mean	20.84	0.69	3.59	3.67	3.64	3.67	3.67	3.68	3.63	3.77	3.66	3.56	2.59
S.D	1.53	0.22	0.96	0.91	0.88	0.77	0.80	0.96	0.57	0.87	0.95	0.89	0.93

N = 283; *p < 0.05, **p < 0.01, neuro stands for neuroticism, agree stands for agreeableness, extra stands for extraversion, consc stands for conscientiousness, open stands for openness to experience, creat stands for creativity, ft stands for frustration tolerance, ne stands for negative emotion, cr stands for cognitive reappraisal, es stands for expressive suppression. mv stands for method variable. The same below.

## Analyses and Results

We used a hierarchical linear regression model to test the hypotheses. Table 1 presents the correlation matrix of the variables.

Table 2 presents the results of our hypotheses testing. Hypothesis 1 predicted that negative emotion would have a negative effect on creativity. Model 2 in Table 2 shows that the coefficient of negative emotion is significant (β = −0.24, *p* < 0.001), so hypothesis 1 is supported. We also found support for Hypothesis 2, which hypothesized a positive effect of students’ frustration tolerance on their creativity (β = 0.21, *p* < 0.001). According to the research of Baron and Kenny (1986) for testing mediating effect, we found in model 1 and 2 that frustration tolerance can partially mediate the relationship between negative emotion and creativity, thus supporting Hypothesis 3. Models 5 includes the interaction effects between negative emotion and cognitive reappraisal, and the results show that cognitive reappraisal negatively moderates the relationship between negative emotion and creativity (β = −0.31, *p* < 0.001), as proposed by Hypothesis 4. Similarly, Models 6 includes the interaction effects between negative emotion and expressive suppression, and the results show that expressive suppression positively moderates the relationship between negative emotion and creativity (β = 0.20, *p* < 0.05), as proposed by Hypothesis 5. Therefore, Hypothesis 4 and 5 are all supported.

**TABLE 2:** The results of hierarchical linear regression (*n* = 283).

	Model 1	Model 2	Model 3	Model 4	Model 5	Model 6
age	0.02 (0.06)	0.02 (0.06)	0.02 (0.06)	−0.01 (0.06)	−0.01 (0.06)	−0.01 (0.06)
gender	0.01 (0.06)	0.01 (0.06)	0.01 (0.06)	0.02 (0.6 = 06)	0.02 (0.06)	0.02 (0.06)
grade	−0.01 (0.06)	−0.01 (0.06)	−0.01 (0.06)	−0.01 (0.06)	−0.01 (0.06)	−0.02 (0.06)
neuro	−0.06 (0.09)	−0.05 (0.09)	−0.07 (0.09)	−0.07 (0.09)	−0.08 (0.09)	−0.07 (0.09)
agree	0.12 (0.46)	0.11 (0.45)	0.09 (0.46)	0.10 (0.45)	0.13 (0.46)	0.10 (0.44)
extra	0.08 (0.53)	0.07 (0.54)	0.10 (0.52)	0.08 (0.51)	0.10 (0.54)	0.11 (0.56)
consc	0.11 (0.74)	0.12 (0.78)	0.10 (0.72)	0.11 (0.74)	0.12 (0.76)	0.13 (0.74)
open	−0.11 (0.30)	−0.12 (0.34)	−0.13 (0.33)	−0.14 (0.37)	−0.11 (0.36)	−0.10 (0.35)
ne		−0.24*** (0.08)	−0.17* (0.08)	−0.19* (0.08)	−0.20* (0.09)	−0.19* (0.08)
ft			0.21*** (0.06)	0.24*** (0.06)	0.20*** (0.06)	0.22*** (0.06)
cr				0.17* (0.08)	0.20* (0.09)	0.21* (0.09)
es				−0.22* (0.10)	−0.18* (0.09)	−0.19* (0.09)
Ne × cr					−0.31*** (0.08)	
Ne × es						0.20* (0.09)
constant	4.03	4.40	4.39	5.07	5.20	4.67
*R* ^2^	0.21	0.23	0.25	0.26	0.28	0.30
Adjusted *R*^2^	0.19	0.20	0.23	0.23	0.25	0.27
Δ*R*^2^	–	0.02	0.02	0.01	0.02	0.02
*F-value*	27.12***	34.39***	35.13***	39.61***	40.77***	41.35***

***p ≤ 0.001, **p ≤ 0.01, *p ≤ 0.05. Two-tailed reported. The values in brackets are standard errors. The results are unstandardized.

**FIGURE 2:**
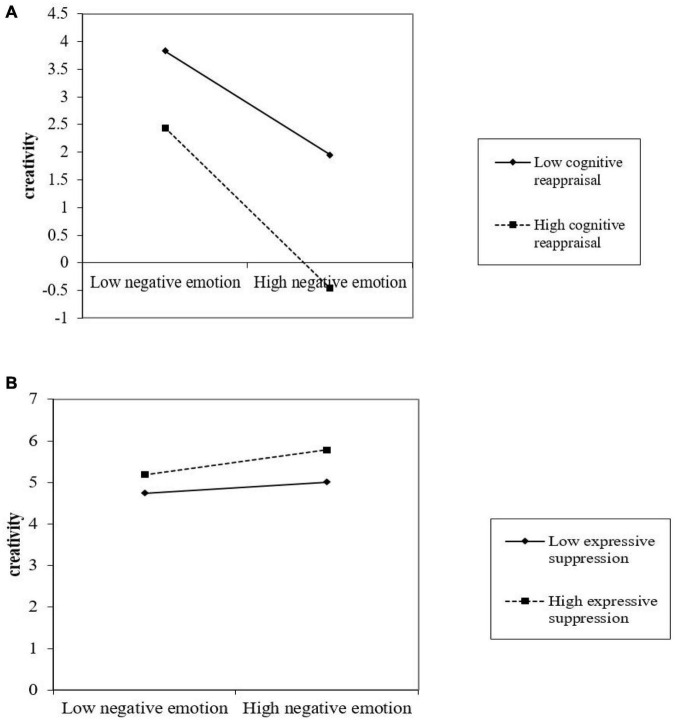
**(A)** Negative emotion × cognitive reappraisal. **(B)** Negative emotion × expressive suppression.

In order to gain a better understanding of their effects, we graphed these interaction effects in Figure 2. Figure 2A shows that students with high cognitive reappraisal had a negative impact on the relationship between negative emotion and creativity, that is to say, the higher the cognitive reappraisal, the weaker the negative impact of negative emotion on creativity. While Figure 2B shows that students with high expressive suppression had a positive impact on the relationship between negative emotion and creativity, that is to say, the higher the expressive suppression, the stronger the negative impact of negative emotion on creativity. Figure 2 provides further evidence for Hypotheses 4 and 5.

## Discussion and Implications

### Discussion and Contributions

In line with the emotive view and cognitive appraisal theory, we integrated the concept of negative emotion, frustration tolerance, and creativity and emotion regulation into a research framework to provide an in-depth understanding of how emotions of student influence creativity in music education and can help student cope with the emotional swings in their college life. Specifically, we found that negative emotion can decrease the creativity while frustration tolerance can facilitate the student’s creativity. Besides, frustration tolerance can mediate the relationship between negative emotion and creativity. Considering the boundary conditions, students’ cognitive reappraisal and expressive suppression, two strategies that represent antecedent- and response-focused ways of regulating emotions (Gross and John, 2003) have significant influence on creativity improvement.

Speaking of the negative effect of negative emotion on creativity, negative emotions can affect an individual’s understanding and integration of information. In terms of mental model, negative emotions can also lead an individual to be more conservative, thus limiting the use of information and reducing the generation of individual creativity (Hershkovitz et al., 2019). In addition, negative emotions can reduce the stimulation of knowledge and make individuals tend to deal with problems in a conservative way, thus reducing the improvement of creativity.

Frustration tolerance, as we all know, is a kind of ability to defeat setbacks and obstacles. Individual with high frustration tolerance is not affected greatly by negative emotions of setbacks, can take the initiative to face setbacks, make good use of their own resources, find solutions and take effective actions. Most of the individuals with high frustration tolerance have strong willpower, believe that they have the ability to overcome difficulties, and will be braver when encountering failure and setbacks. For college students, frustration tolerance can solve the anxiety and confusion caused by negative emotions, so that they can fight back in adversity and improve their creativity.

Because cognitive reappraisal typically preempts extreme emotions (De Cock et al., 2019), and stimulates students to generate and discuss options for improving behaviors, we expected a positive moderating impact of cognitive reappraisal on the relationship between negative emotion and creativity, which can make it easy for students to use an antecedent-focused emotion regulation strategy effectively to anticipate what may happen in future and adjust their negative emotions by judging the emotional impact. It is simple for students to create a stable experience using cognitive reappraisal, which can keep them out of bad situations and perceive negative events accurately and activate problem-focused coping mechanisms. The negative effect of negative emotion on creativity is reversed principally by cognitive reappraisal.

For expressive suppression, we hypothesized and found a positive moderating effect on the relationship between negative emotion and creativity. As expressive suppression leads to emotional dissonance and lower satisfaction, which can cause students to give up the current work, lower the tendency for improvisation and quit discussion and communication. Students with negative emotions may suffer from expressive suppression, which may undermine student’s chances of showing amiableness and talkativeness when facing frustration. Then students cannot improve creativity under the interaction of negative emotions and expressive suppression.

This study has several theoretical contributions. First, the main contribution is to the literature on creativity, where an amount of research argued that creativity is influenced by personality, Big Five factors, inspiration, training and learning, collaboration, information and communication technologies and psychology (Stolaki and Economides, 2018; Huang, 2019; Lai et al., 2019; Liu et al., 2020; Hsia et al., 2021; Rahimi and Shute, 2021; Roth et al., 2021), but little attention is paid to the impact of emotions (Falavarjani and Yeh, 2018). This study is one of the cutting-edge research to investigate how students in music education can deal with creativity to influence their performance. Studies of college student’s creativity in emotive context are scarce, tend to overlook some important influential factors and outcomes (Dumas et al., 2021). Second, researchers found that negative emotion can cause unpleasant or bad outcomes, thus affecting the creative ideas or thinking (Guan et al., 2021). There is little research on how to change the adverse effects of negative emotions and the mechanism has not been effectively explored. We introduced frustration tolerance as a mediator to guide the positive transformation of negative emotions for college student, especially in music education. This study opens the black box between negative emotions and creativity and elaborate the path for students’ creativity improvement under negative emotions. Last but not the least, our study contribute to the literature of emotion regulation. Previous studies focus on the direct influence of emotion regulation on negative emotions and creativity (in the perspective of psychology), its contingency effect lack enough attention. Our study focuses on the under-studied contingency effect of emotion regulation from two different views, one is antecedent-focused way (cognitive reappraisal) and the other is response-focused way (expressive suppression). By doing so, we address recent calls of researchers to investigate how students can influence their creativity to the benefit of their emotion regulation (e.g., Hershkovitz et al., 2019; Holdhus, 2019). Most remarkably, we also make contributions for cognitive appraisal theory in explaining how to cope with emotive events by find useful strategies to regulate negative emotions in music education.

Our study also has several practical implications. The first is for students, who should be aware that their emotions, and more importantly how they habitually regulate them, may have a significant impact on their daily behaviors and their creativity. Students should strengthen their frustration tolerance when facing setbacks or in bad mood, especially in music creation. In addition, considering the negative effect of negative emotion, colleges and teachers should pay close attention to the students’ psychological state and encourage them to establish a correct outlook on life, be persistent when confronted with problems, and train them to be positive, innovative and creative. Most importantly, students should cultivate their tolerance to face difficulties and frustration, and creativity for production when facing negative mood. To some extent, cultivation of tolerance and creativity and control of emotion can be parallel.

### Limitations and Future Research Directions

Our results suggest that antecedent- and response-focused emotion regulation has different impact on the relationship between negative emotion and creativity. But we know that cognitive reappraisal and expressive suppression are only two strategies out of five. Future research might investigate whether other types of emotion regulation strategies have similar effects on the above relationship. For example, future studies might examine emotion regulation actions at the stages of situation selection, situation modification, and attentional deployment to determine whether these forms of antecedent-focused emotion regulation relate to creativity similarly as cognitive reappraisal and expressive suppression do.

Another limitation of our study is that all data are collected in a certain period, which incurred cross sectional problem. Because we use cross-sectional data to make the analyses, the direction of causality cannot be fully verified. Emotion regulation changes over time, so do frustration tolerance and students’ creativity (Bardeen et al., 2017). Although we argue that frustration tolerance is conducive to students’ creativity, it is possible to produce alternative results. For example, a student’s high level of frustration tolerance might result in bad creativity because of the intensity of tolerance, which can cause more negative blowout of emotion and bring negative results. Future research should focus on the longitudinal view and study the above relationships with longitudinal data to investigate the possible law.

Finally, although the results provide support for our hypotheses, we acknowledge that our focus on data from Northeast of China, a relatively under-developed area in China, raises questions about the generalizability of our study beyond regions. Northeast of China is full of several special characteristics, including laggard economic development, solidified thought and weak creativity, which may exert influences on students’ thinking and behavior. Future research should broaden the areas for data collection to evaluate the external validity of our model by testing it in different areas and compare the differences among different areas to get a general view.

## Data Availability Statement

The original contributions presented in the study are included in the article/Supplementary Material, further inquiries can be directed to the corresponding author.

## Author Contributions

LW designed the model and collected the data. NJ analyzed the data. LW wrote the parts 1, 2, and 5, NJ finished the other parts. Both authors contributed to the article and approved the submitted version.

## Conflict of Interest

The authors declare that the research was conducted in the absence of any commercial or financial relationships that could be construed as a potential conflict of interest.

## Publisher’s Note

All claims expressed in this article are solely those of the authors and do not necessarily represent those of their affiliated organizations, or those of the publisher, the editors and the reviewers. Any product that may be evaluated in this article, or claim that may be made by its manufacturer, is not guaranteed or endorsed by the publisher.
