# Supplementary material for: Managing Students’ Creativity in Music Education – The Mediating Role of Frustration Tolerance and Moderating Role of Emotion Regulation
Source: Front Psychol. 2022 Apr 13;13:843531. doi: 10.3389/fpsyg.2022.843531 (PMC9045781; doi:10.3389/fpsyg.2022.843531)
Supplement: Supplementary file 1 [file Table_1.pdf]

Supplementary Appendix Material

Supplementary Appendix Table 1.

Creativity measurement items

Creativity (Torrance and Myers ,1970)	Originality(.820)	The degree of innovation in a method of solving a problem
	constructive diversity(.825)	the number and diversity of methods proposed to solve problems
	analytical power(.853)	the degree of details of the method of solving problem
	Feasibility(.782)	the degree to which the method proposed to solve the problem can be applied in real life

Source: Torrance, E. P., & Myers, R. （1970） . Creative Learning and Teaching. New York: Dodd, Mead, and Company: Inc. The values in brackets are factor loading. The same below.

Supplementary Appendix Table 2.

Frustration tolerance measurement items	
Frustration tolerance (Clifford, 1984)	1. I will keep on working until the assigned work is completed. I won't give up halfway.(.874)<br>2. If I don't understand something, I will consult others, the Internet or books.(.866)<br>3. If I do not do a good job on the test, I will find ways to improve and try again next time. (.822)<br>4. I feel excited about taking part in challenging activities. (***deleted***)<br>5. I think no matter how difficult the problem is, it will be solved. Don't worry about it. (.633)<br>6. I find it exhilarating to think or solve problems. (.874)<br>7. I am willing to try a new kind of work. (.866)<br>8. I'm willing to do things I'm not good at. (.822)<br>9. I take the initiative to learn new things.(.840)<br>10. When misunderstood by classmates or teachers, I will try my best to explain clearly.(.798)<br>11. I will take the initiative to communicate with my friends after a quarrel. (.810)<br>12. When communication with my parents fails, I will try another way.(.825)<br>13. When I quarrel with my friends, I will take the initiative to communicate with them first.(.764)<br>14. When I am criticized by others, I deal with it rationally. I don't feel sad or angry all the time.(.840)<br>15. It will be fun to try to adapt to a different kind of people.(.798)<br>16. If a friend needs improvement, I am willing to talk to him.(.810)<br>17. I actively ask people if there is anything I can do to improve myself.(.825)<br>18. I take the initiative to meet new people even though I may get rejected.(***deleted***)

Source: Clifford, M.M.(1984).Thoughts on a theory of constructive failure. Educational Psychologist, 19, 108-120.

Supplementary Appendix Table 3.

Negative emotion measurement items	
Negative emotion<br>Fisher (1997)	1. Depressed (.845)<br>2. frustrated (.822)<br>3. Angry (.730)<br>4. Disgusted (.730)<br>5. Unhappy (.759)<br>6. Disappointed (.746)<br>7. Embarrassed (.744)<br>8. Worried (.534)

Source: Fisher, C. D. (1997). Emotions at work: what do people feel and how should we measure it? School of Business Discussion Papers. Paper 63. http://epublications.bond.edu.au/discussion_papers/63

Supplementary Appendix Table 4.

Emotion regulation measurement items

Emotion regulation	Gross and John (2003).
Cognitive reappraisal	1. I control my emotions by changing the way I think about the situation I'm in.(.797)<br>2. 2. When I want to feel less negative emotion, I change the way I'm thinking about the situation.(.814)<br>3. When I want to feel more positive emotion, I change the way I'm thinking about the situation.(.814)<br>4. When I want to feel more positive emotion (such as joy or amusement), I change what I'm thinking about.(.781)<br>5. When I want to feel less negative emotion (such as sadness or anger), I change what I'm thinking about. (.781)<br>6. When I'm faced with a stressful situation, I make myself think about it in a way that helps me stay calm. (.773)
Expressive suppression	1. I control my emotions by not expressing them.(.783)<br>2. When I am feeling negative emotions, I make sure not to express them.(.792)<br>3. I keep my emotions to myself.(.774)<br>4. When I am feeling positive emotions, I am careful not to express them.(.803)

Source: Gross, J.J., John, O.P., 2003. Individual differences in two emotion regulation processes: implications for affect, relationships, and well-being. J. Pers. Soc. Psychol. 85(2), 348.

Supplementary Appendix Table 5.

Big Five Factors measurement items

Please clarity: I see myself as someone who…

Big Five Factors John et al. (1991)	1.Is talkative (.733)<br>2.Tends to find fault with others(.829)<br>3.Does a thorough job***(deleted)***<br>4.Is depressed, blue(.896)<br>5.Is original, come up with new ideas(.828)<br>6.Is reserved(.869)<br>7.Is helpful and unselfish with others(.805)<br>8.Can be somewhat careless (.840)<br>9.Is relaxed, handles stress well(.947)<br>10.Is curious about many different things(.803)<br>11.Is full of energy(.775)<br>12.Start quarrels with others(.805)<br>13.Is a reliable worker(.808)<br>14.Can be tense(.754)<br>15.Is ingenious, a deep thinker(.805)	16.Generates a lot of enthusiasm(.848)<br>17.Has a forgiving nature(.828)<br>18.Tends to be organized(.813)<br>19.Worried a lot ***(deleted)***<br>20.Has an active imagination(.823)<br>21.Tends to be quiet(.850)<br>22.Is generally trusting(.772)<br>23.Tends to be lazy(.778)<br>24.Is emotionally stable, not easily upset(.602)<br>25.Is inventive(.762)<br>26.Has a assertive personality(.794)<br>27.Can be cold and aloof(.805)<br>28.Perseveres until the task is finished(.752)<br>29.Can be moody(.806)<br>30.Value artistic, aesthetic experience(.799)	31.Is sometimes shy, inhibited(.767)<br>32.Is considerate and kind to almost everyone(.805)<br>33.Does things efficiently(.812)<br>34.Remains calm in tense situations (.814)<br>35.Prefers work that is routine(.801)<br>36.Is outgoing, sociable(.848)<br>37.Is sometimes rude to others(.768)<br>38.Makes plans and follows with them(.776)<br>39.Get nervous easily (.758)<br>40.Likes to reflect, play with ideas(.765)<br>41.Has few artistic ideas(.793)<br>42.Likes cooperate with others(.818)<br>43.Is easily detracted(.807)<br>44.Is sophisticated in art, music or literature***(deleted)***

Source: John, O.P., Donahue, E.M., Kentle, R.L., 1991. The Big Five Inventory: Versions 4a and 54, Institute of Personality and Social Research. University of California, Berkeley, CA.

Note: there are 44 measurement items to evaluate Big Five Factors. Extraversion includes items 1,6,11,16,21,26,31,36; agreeableness includes items 2,7,12,17,22,27,32,37,42; conscientiousness includes items 3,8,13,18,23,28,33,38,43, neuroticism includes items 4,9,14,19,24,29,34,39 and openness to experience includes items 5,10,15,20,25,30,35,40,41,44.
